# Is There a Link between Wheezing in Early Childhood and Adverse Birth Outcomes? A Systematic Review

**DOI:** 10.3390/ijerph6112752

**Published:** 2009-11-03

**Authors:** Evridiki Patelarou, Maria Chochlidaki, Victoria Vivilaki, Hero Brokalaki

**Affiliations:** 1 University Hospital of Heraklion, Crete, 71414 Greece; 2 Independent Researcher, Crete, Heraklion, 71500 Greece; E-Mail: maria_choch@yahoo.gr; 3 Department of Midwifery, Technological Educational Institution, Athens, 12210 Greece; E-Mail: v_vivilaki@yahoo.co.uk; 4 Faculty of Nursing, University of Athens, 11527 Greece; E-Mail: heropan@nurs.uoa.gr

**Keywords:** adverse birth outcomes, low birth weight, preterm, wheezing, respiratory disorder, asthma

## Abstract

We aimed to provide a summary of the existing published knowledge on the association between adverse birth outcomes and the development of wheezing during the first two years of life. We carried out a systematic review of epidemiological studies within the MEDLINE database. Epidemiological studies on human subjects, published in English, were included in the review. A comprehensive literature search yielded 72 studies for further consideration. Following the application of the eligibility criteria we identified nine studies. A positive association and an excess risk of wheezing during the first two years of life were revealed for adverse birth outcomes.

## 1. Introduction

A number of maternal and lifestyle factors have been shown to be associated with the occurrence of wheezing or diagnosed asthma in childhood and adolescence, such as maternal age [1], maternal smoking before birth and exposure to secondhand smoke (SHS) [2,3]. Maternal smoking, low maternal age and early bottle feeding are also associated with lower respiratory tract illness in the first 2 years of life, along with environmental exposures such as dampness [4,5] which, in turn, are linked with the development of asthma in childhood [6]. Consequently this evidence suggests that exposures occurring during pregnancy and early childhood can influence the risk of respiratory signs, such as wheezing in early childhood, or asthma later on in life.

Furthermore, low birth weight or very low birth weight infants are known to develop decreased respiratory function and have been found to be at an increased risk for the development of chronic respiratory symptoms during childhood [7–10]. Prematurely born children are known to have more respiratory symptoms during their first years of life than children born full-term [10–13] and their lung function at school age has been shown to be significantly reduced [14–17] especially among the children who had bronchopulmonary dysplasia at birth [14,18,19].Taking the above into account both prematurity and low birth weight are considered significant risk factors for the development of childhood wheezing and asthma, even if the aetiology and the mechanism by which adverse birth outcomes predispose to wheezing is not known [20–22]. Although the above causal relationships are of significant importance to both pediatrics and public health, the international literature remarkably lacks information on the respiratory symptoms that are experienced by infants born growth restricted or premature. Taking the above into account, this systematic review aims to summarize the existing published scientific knowledge regarding the association between adverse birth outcomes and the development of wheezing during the first two years of life.

## 2. Methodology

A systematic review of the existing literature on adverse birth outcomes related and wheezing was carried out. We posed the following review question: “Given the existing epidemiological evidence, is there a link between adverse birth outcomes and the occurrence of wheezing during the first two years of life?”. We drew up a review protocol in advance following standards outlined in the MOOSE Guidelines for Meta-Analyses and Systematic Reviews of Observational Studies [23]. We carried out a systematic, comprehensive bibliographic search using the US National Library of Medicine Medline database for the years 1990–2009, using the PubMed interface due to its free access to abstracts.

Search terms used were chosen from the USNLM Institutes of Health list of Medical Subject Headings (MeSH) for 2007. These were: “Infant, Low Birth Weight” OR “Infant, Very Low Birth Weight” OR “Premature Birth” OR “Fetal Growth Retardation” OR “Infant, Extremely Low Birth Weight” AND “Respiratory Sounds”, OR “Signs and Symptoms, Respiratory” OR “Wheezing”. Although not officially MeSH terms, “Wheezing” and “Small for gestational age” was also added as key terms so as to broaden the scope of the search. Retrieved studies were checked against a list of eligibility criteria, while the references of each retrieved study were also checked by hand for additional studies that met the eligibility criteria.

We defined *a priori* eligibility criteria to restrict the studies included. Studies were only included if they referred to humans, were published in English after 1990, were epidemiological studies (of any study design) and they examined the presence of wheezing up to two years old. Studies not meeting these criteria were excluded from the review. Data were extracted systematically from each included study by two researchers separately using a standardized data extraction form. The following data were extracted from each study: study main characteristics, study population, study topic, and measures of effect and confidence intervals for each outcome.

## 3. Results

**Figure 1.**
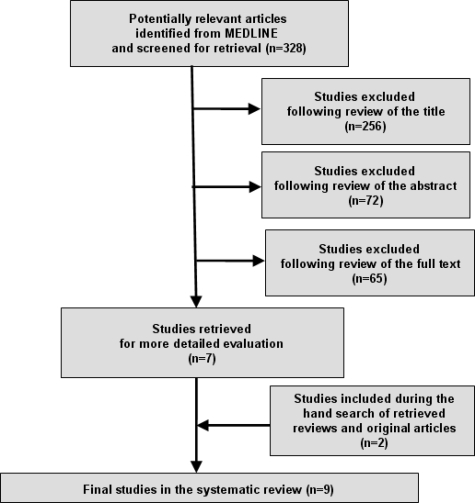
Literature search and strategy outcomes.

Figure 1 demonstrates the numbers of studies identified and selected/excluded in each phase of the search. Manual searching of bibliographies provided some additional studies (two) that met the broad eligibility criteria. Ultimately, nine studies were deemed suitable for inclusion in the review although one study included two sub-studies [24] and as a result we finally considered ten studies as the final number included in the systematic review.

The main characteristics of the studies included in the analysis are given in Table 1. The systematic review included nine prospective cohort studies [24–32] and one case control study [24]. Four studies were conducted in the USA, four in the UK, one in Italy and one in Poland.

Table 2 summarizes the main findings of the ten studies included in the systematic review. Four studies reported their results as odds ratios [28,29,31,32] one as relative risk ratio [25] and five as prevalence [24,26,30]. There were also some disparities in the definitions of adverse birth outcomes between studies. Two studies examined LBW which is commonly defined as birthweight <2500 g. PD was examined by six studies and was generally defined as a birth <37 weeks of gestation [24,29], although Lewis *et al.* [32] used gestational age <36 weeks and Holditch-Davis *et al.* [27] gestational age <35 weeks as the cut off for PD definition. The definition of VPD varied between the three studies by using as a cut off the <29 gestational weeks [30], the <34 gestational weeks [32] and the <33 gestational weeks [28], respectively. VLBW was studied in four studies [24,26,32]. Halterman *et al.* [26] used the criterion of birth weight <1,500 g, Lewis *et al.* [32] defined VLBW as birth weight <2,000 g while the definition for VLBW by Grenough *et al.* [24] was not available. One study examined the infant length at birth as an outcome [25] while term low birth weight and small for gestational age was not studied in any of the previously mentioned studies.

All studies examined wheezing as an outcome although the time period of wheezing presence was different. Four studies examined wheezing at first year of life [24,26,30], four studies examined wheezing up to two years of life [25,27,29,31], one study examined wheezing up to five years old [32] and one included children with a mean age of 2.2 years old [28] (the last two studies remained in the review due to the fact that they also examined wheezing presence at the age of 2 years old).

Four studies measured the outcome as an odds ratio [28,29,31,32]. Galli *et al.* [31] underlined an excess risk of wheezing presence for LBW infants (OR 11.88 95%CI 6.01–23.47) and Kumar *et al.* [28] underlined the clear association of wheezing and both preterm and very preterm delivery (OR 1.7 95%CI 1.2–2.6 and OR 2.7 95%CI 1.3–5.5, respectively). Additionally, Lewis *et al.* [32] although referring to the wheezing presence up to five years old reported a risk for LBW infants but it was not statistically significant (OR 1.22 95%CI 0.96–1.54). No statistically significant associations were also revealed for wheezing and PD, VPD, VLBW by Lewis *et al.* [32]. Taveras *et al.* [29] also did not find any correlation between PD and wheezing at first two years of life. Additionally, Jedrychowski *et al.* [25] reported results as a relative risk ratio but did not reveal any correlation between infant length at birth and wheezing presence.

The prevalence of wheezing was also estimated by five studies [24,26,27,30]. Grenough *et al.* [24] in the two separate studies reported similar findings for wheezing prevalence during the first year of life (65% in the case-control and 53% in the cohort study) for infants that were both PD and VLBW. Additionally, VLBW infants were found to present wheezing, cough or heavy breathing in a percentage of 26% [26] while Grenough *et al.* [30] reported 42% prevalence of wheezing among VPD infants. Finally, Holditch-Davis *et al.* [27] reported wheezing at different ages (2, 6, 9, 13, 18 and 22 months) in a population of preterm infants with wheezing prevalence ranging from 8% (at two months) up to 26% (at 18 months).

## 4. Discussion

As indicated through this systematic review, we have gathered the existing epidemiological evidence in order to examine the possible association between adverse birth outcomes and the development of wheezing during early childhood. Furthermore we identified a positive association between adverse birth outcomes such as LBW, VLBW, PD, VPD and the development of wheezing during early childhood. No studies examining the association of term low birth weight and small for gestational age infants with wheezing were revealed and thus these outcomes were not further investigated into.

Considerable variation in the prevalence of wheezing has been observed in previous studies. A prevalence increase has been noticed between countries and over time specifically from the 1970s up to the early 1990s [33]. These differences can be partially explained by the studies’ geographical variation and the deficiency of a common and strict definition of wheezing [33]. Prevalence of wheezing symptoms in children has been studied extensively while there is gap in the literature concerning wheezing prevalence during early childhood.

There are many potential causes of wheezing including genetic/familial or environmental factors, and viral respiratory infections [34]. Generally, exposure to a number of toxicants during lung development has the potential to significantly affect the function of the respiratory system of children [35]. Among other factors, maternal malnutrition during pregnancy, smoking both active and passive, chemicals and particles are only some of the potential hazards. A strong link also exists between the prenatal PM2.5 exposure, moldy/ damp house, maternal atopy and presence of older siblings with the severity of wheezing and respiratory illness [25]. However, low birth weight, preterm birth, low maternal age, household size, maternal smoking, infant feeding practices, and socioeconomic status are all interrelated and as a result it is difficult to distinguish which of these factors consist the independent determinants of wheezing during early childhood [32].

The majority of children who develop wheeze in early childhood are free of symptoms by adolescence or early adulthood [36,37]. In addition, it is not clear whether wheezing that resolves in early childhood and wheezing which persists into adolescence represent the same disease, or they consist manifestations of fundamentally separate disease processes [38,39]. Wheezing is often followed up with asthma and lung function abnormalities since a significant proportion of children who develop asthma wheezed in early life [28]. Abnormalities in lung function after early childhood wheezing seem to continue at least until teenage years. Most children with early childhood wheezing outgrow their symptoms during school years, but asthma relapses are common in young adults as childhood wheezing is associated with permanent changes in the airways that continue until adulthood [40]. Further investigation is needed so as to conclude whether an increased prevalence of wheezing during early childhood is correlated with an increased prevalence of asthma and related health outcomes in late childhood and adolescence.

Early life events are important since the origin of airway abnormalities occurs early in infancy. Prospective birth cohort studies clarify the incidence of an illness by evaluating the risk factors and the possible confounders and/or modifiers related to the disease. This kind of studies may allow us to shed light on the primary factors that initiate wheezing and its correlation to long term implications such as chronic obstructive lung disease.

Conclusively, this study area is of high importance due to the fact that long term implications are unknown. It is therefore desirable to determine the correlation between adverse birth outcomes and wheezing during early childhood and identify whether there are preventable or treatable risk factors. For the purpose of this study, review was restricted to wheezing as a health outcome while other important causes of wheezing during early childhood other than adverse birth outcomes (e.g., genetic/familial or environmental factors) were not examined. As mentioned above, well designed epidemiological studies which will evaluate the relevant confounders and possible exposures and risk factors during pregnancy are needed. This estimation of summary should be considered by epidemiologists, health care specialists and research community as the most interesting areas for further research work.

## Figures and Tables

**Table 1.** Summary of characteristics of studies included in the systematic review.

**Authors**	**Main study characteristics**	**Study population**	**Study Topic**
Jedrychowski *et al.* (2009)	Poland 2003–2004 Prospective cohort study	468 infants	Assess possible association of early persistent wheezing with the length of the baby at birth.
Halterman *et al.* (2009)	New York, USA 2003–2006 Prospective cohort study	124 VLBW	Identify the environmental exposures and respiratory morbidity among VLBW infants and determine the association between them.
Holditch-Davis et al.* (2008)	USA Prospective cohort study	113 PD	Identify environmental and medical factors related to the development of wheezing in prematurely born children over the first 27 months after term.
Kumar *et al.* (2008)	Boston, USA Initiated in 1998 (ongoing) Prospective cohort study	1,096 infants	Evaluate the association between prematurity and wheezing accounting for the presence of perinatal chorioamnionitis.
Taveras et al.* (2006)	Boston, USA Prospective cohort study	1,372 infants	Examine the associations of fetal growth and length of gestation with asthma-related outcomes by age 2 years.
Greenough et al.* (2005)	London, UK Prospective cohort study	492 PD	Identify the occurrence of respiratory morbidity during infancy after preterm birth and identify the risk factors.
Galli *et al.* (2003)	Florence, Italy 1992–1998 Prospective cohort study	1,780 infants	Wheezing illness in HIV-1 infected infants.
Lewis *et al.* (1995)	Nottingham, UK 1970 Prospective cohort study	15,712 Infants	Study of risk factors for early and persistent wheezing until age of five years old.
Greenough et al.* (1990a)	London, UK Case- control	PD and VLBW	Identify the respiratory symptoms in the first year of life following preterm delivery.
Greenough et al.* (1990b)	London, UK Prospective cohort study	PD and VLBW	Identify the respiratory symptoms in the first year of life following preterm delivery.

* No information regarding time period for data collection.

**Table 2.** Main findings of studies included in the systematic review.

**Study**	**Birth outcomes investigated**	**Infants’ age**	**Outcome definition**	**Outcome measure**	**Measures of effect (95% confidence intervals)**
Jedrychowski *et al.* (2009)	Infant length at birth (n *=* 468)	2 years old	Infant length as a continuous variable (cm)	Relative risk ratio	Transient early wheezing Length at birth 1.03 (0.92–1.15) Transient late wheezing Length at birth 0.92 (0.82–1.02) Persistent wheezing Length at birth 0.88 (0.77–1.00)
Halterman *et al.* (2009)	VLBW (n *=* 124)	1 years old	<1500 g	Prevalence	At 12 months 26% of infants experienced symptoms of wheeze, cough, or heavy breathing
Holditch-Davis *et al.* (2008)	PD (n *=* 113)	2, 6, 9, 13, 18, 22 months	GA < 35	Prevalence	Wheezing prevalence: At 2 months Mild wheezing 8.2% Significant wheezing 8.2% At 6 months Mild wheezing 17.4% Significant wheezing 13.3% At 9 months Mild wheezing 22.2% Significant wheezing 14.4% At 13 months Mild wheezing 17.1 % Significant wheezing 22.7% At 18 months Mild wheezing 20.2% Significant wheezing 25.5% At 22 months Mild wheezing 17.3% Significant wheezing 18.5%
Kumar *et al.* (2008)	PD (n *=* 178) VPD (n *=* 63)	Mean age 2.2 years old	GA33–36.9 GA < 33	Odds ratio	PD 1.7 95% CI (1.2–2.6) VPD 2.7 95% CI (1.3–5.5)
Taveras *et al.* (2006)	PD (n *=* 73)	2 years old	GA < 37	Odds ratio	Any wheezing PD 1.25 95% CI (0.69–2.28) Persistent wheezing PD 1.19 95% CI (0.53–2.66)
Greenough *et al.* (2005)	VPD (n *=* 492)	1 year old	GA < 29	Prevalence	Wheezing prevalence: At 6 months 40% At 12 months 42% Both at 6 and 12 months 20%
Galli *et al.* (2003)	LBW (n *=* 160)	2 years old	LBW ≤ 2,500g	Odds ratio	11.88 (6.01–23.47)
Lewis *et al.* (1995)	LBW (n *=* 599) VLBW (n *=* 178) PD (n *=* 464) VPD (n *=* 154)	Up to 5 years old	LBW ≤ 2,500g VLBW ≤ 2,000g PD < 36 VPD < 34	Odds ratio	LBW 1.22 95% CI (0.96–1.54) VLBW 1.34 95% CI (0.93–1.92) PD 1.02 95% CI (0.77–1.35) VPD 1.33 95% CI (0.92–1.92)
Greenough et al.* (1990a)	PD and VLBW	1 year old	PD < 36 VLBW	Prevalence	Wheeze or wheeze & cough 65% in PD and VLBW infants vs 33% in control group
Greenough et al.* (1990b)	PD and VLBW	1 year old	PD < 36 VLBW	Prevalence	Wheeze or wheeze & cough 53% PD and VLBW infants

* The exact number of cases for PD and VLBW was not available.
